# IMPLEMENTATION OF ENHANCED RECOVERY AFTER COLORECTAL SURGERY (ERAS) PROTOCOL: INITIAL RESULTS OF THE FIRST BRAZILIAN EXPERIENCE

**DOI:** 10.1590/0102-672020180001e1419

**Published:** 2019-02-07

**Authors:** Uirá Fernandes TEIXEIRA, Paulo Roberto Ott FONTES, Cristiane Weckerle Nazareth CONCEIÇÃO, Carlos Alberto Teixeira FARIAS, Daieni FERNANDES, Ingrid Petroni EWALD, Luciano VITOLA, Florentino Fernandes MENDES

**Affiliations:** 1 Department of Surgery, Federal University of Health Sciences of Porto Alegre and Santa Casa de Misericórdia of Porto Alegre; 2 Hospital Quality Sector; 3 Anesthesiology Service; 4 Nutrition Service of Santa Casa de Misericórdia of Porto Alegre; 5 Department of Anesthesiology, Santa Casa de Misericórdia of Porto Alegre/Federal University of Health Sciences of Porto Alegre), Porto Alegre, RS, Brazil.

**Keywords:** Colorectal surger, Postoperative car, Length of sta, Cirurgia colorreta, Cuidados pós-operatório, Tempo de internação

## Abstract

**Background:**

Guidelines for enhanced recovery after surgery have their bases in colonic surgery, through the first protocols published in 2012. Since then, this practice has spread throughout the world, mainly due to improvements in surgical outcomes associated with resource savings.

**Aim:**

To analyze the first prospective results after the implementation of the guidelines.

**Methods:**

Were retrospectively analyzed 48 patients operated in the institution prior to the standardization. This group was then compared with a series of 25 patients operated consecutively after the guidelines were implemented.

**Results:**

With a 68.6% compliance rate, hospital length of stay (p=0.002), use of abdominal drains (p<0.001) and mechanical bowel preparation (p<0.001) were reduced. Mortality rates, anastomotic fistula, abdominal abscesses and reoperations were also reduced, but without statistical significance.

**Conclusion:**

Enhanced recovery after surgery protocols benefit patients care, resulting in better outcomes and possibly resource savings. Even with some limitations, its implementation is feasible in the Brazilian Public Health System.

## RESUMO

**Racional:**

Os protocolos de recuperação otimizada após as operações têm as suas bases na cirurgia colônica, através das primeiras diretrizes publicadas em 2012. Desde então, tal prática difundiu-se pelo mundo, principalmente em virtude de melhorias nos resultados cirúrgicos associadas à economia de recursos.

**Objetivo:**

Apresentar os primeiros resultados prospectivos após a implementação das novas medidas.

**Métodos:**

Foram analisados de forma retrospectiva 48 pacientes operados na instituição previamente à aplicação do protocolo. Esse grupo foi então comparado com uma série de 25 pacientes operados de forma consecutiva após a implementação das diretrizes.

**Resultados:**

Com taxa de adesão de 68.6% às medidas propostas, observou-se redução do tempo de internação hospitalar (p=0.002), do uso de drenos abdominais (p<0.001) e do preparo mecânico do cólon (p<0.001). As taxas de mortalidade, de fístula da anastomose, de abscessos abdominais e de reoperações também foram reduzidas, porém sem significância estatística.

**Conclusão:**

A adesão às medidas recomendadas no protocolo é benéfica para pacientes e equipe de assistência, acarretando em melhores resultados e possível economia de recursos. Mesmo com algumas limitações, a sua implementação é factível no Sistema Único de Saúde Brasileiro.

## INTRODUCTION

The creation of the term ERAS (acronym for Enhanced Recovery After Surgery) and the basis for its development emerged in 2001, in London, when a group of European surgeons met to develop guidelines for perioperative management based on evidence from literature^13^. At that time, H. Kehlet had already published a paper reporting the possibility of early hospital discharge in patients that underwent sigmoid colon resections, as opposed to the current scenario of the period^10^. After the creation of an international medical society - ERAS Society - and the publication of the first recommendations dealing with colonic surgery by Gustafsson et al. in 2012^7^, this new concept quickly aroused the interest of the international medical community. The proposal of reduction of surgical stress, maintenance of physiological functions and optimized recovery was materialized in a protocol with 23 items, encompassing the three phases of the surgical act itself: pre, intra and postoperative (Table 1).

**TABLE 1:** Highlights of ERAS protocol

ERAS Protocol	
1.	Preadmission information, education and counseling
2.	Preoperative optimization (stop smoking and alcohol consumption)
3.	Preoperative bowel preparation
4.	Preoperative fasting
5.	Preoperative carbohydrate treatment
6.	Preanesthetic medication
7.	Prophylaxis against thromboembolism
8.	Antimicrobial prophylaxis and skin preparation
9.	Perioperative fluid management
10.	Laparoscopy and modifications of surgical access
11.	Standard anesthetic protocol
12.	Reduction of opioid use
13.	Postoperative nausea and vomiting prophylaxis
14.	Nasogastric intubation
15.	Preventing intraoperative hypothermia
16.	Drainage of the peritoneal cavity after colonic anastomosis
17.	Urinary drainage
18.	Prevention of postoperative ileus
19.	Postoperative analgesia
20.	Perioperative nutritional care
21.	Perioperative control of glucose
22.	Early mobilization
23.	Audit

Colorectal surgery represents a vast field, encompassing complex procedures. It is surrounded by dogmas that begin in the preoperative preparation, passing through intraoperative actions that have been historically reproduced between generations of surgeons, and culminating with imposed restrictions on the patients in the postoperative period, many of them lacking scientific evidence ^2^ ^,^ ^14^ ^,^ ^20^. Perhaps this is why it was chosen for the implementation of ERAS protocols, a paradigm shift that has greatly contributed to patient recovery^13^.

Several studies have shown satisfactory outcomes with the development of ERAS protocols in their institutions^4^ ^,^ ^5^ ^,^ ^16^. In 2016, the ERAS team of Porto Alegre, Porto Alegre, RS, Brazil, completed the training stages and initiated the implementation of these guidelines in the assistance of patients of the public health system. 

Thus, the present paper aims to report this first Brazilian experience applied to colorectal surgery.

## METHODS

The implementation of the protocol had two phases.

The first one was the retrospective evaluation of 50 patients operated in the institution’s public health system, using medical records data, from January to June 2016, submitted to elective colorectal surgery (group 1). Two patients that were operated on emergency situation were excluded from evaluation, thus leaving 48 patients for analysis.

The second phase took place between September 2016 and March 2017, and represents the implementation of the protocol itself. There were prospectively evaluated 25 consecutively operated patients by the same digestive surgery team (group 2). Table 2 shows the study groups.

**TABLE 2:** Comparison between groups

	BEFORE ERAS (48)	ERAS (25)	
Age (years)	60 (20-85)	62 (36-81)	p=0.62
Gender (%)			p=0.32
Male	28 (58%)	14 (56%)	
Female	20 (42%)	11 (44%)	
Anatomical Site (%)			p=0.24
Right Colon	20 (42%)	10 (40%)	
Left Colon	28 (58%)	13 (52%)	
Laparoscopic (%)			p=0.12
Yes	5 (10.4%)	5 (20%)	
No	43 (89.6%)	20 (80%)	
Prophylactic drainage (%)			p<0.001
Yes	33 (68.8%)	4 (16%)	
No	15 (31.2%)	21 (84%)	
Mechanical bowel preparation (%)			p<0.001
Yes	42 (87.5%)	4 (16%)	
No	6 (12.5%)	21 (84%)	

Data were entered in the database of the platform provided by the ERAS Society, with the final outcome being the 30-day mortality rate. The results were generated by the audit platform itself. 

### Statistical analysis

It was performed in the SPSS program version 22.0.0, through the chi-square test of homogeneity and Mann-Whitney test, the latter for comparison of hospitalization times. A level of significance of 5% was used.

## RESULTS

Table 3 shows the results for both groups. It is observed that compliance rate to ERAS protocols in group 1 was 19.6% and 68.6% in group 2. 

**TABLE 3:** Outcomes: comparison between groups

	BEFORE ERAS (48)	ERAS (25)	
Length of hospital stay (days)	11.5 (4-38)	8 (3-26)	p=0.002
Abscess (%)			p=0.43
Yes	4 (8.3%)	1 (4%)	
No	44 (91.7%)	24 (96%)	
Fistula (%)			p=0.23
Yes	6 (12.5%)	1 (4%)	
No	42 (87.5%)	24 (96%)	
Reoperation (%)			p=0.43
Yes	6 (12.5%)	2 (8%)	
No	42 (87.5%)	23 (92%)	
Mortality Rate (%)			p=0.57
Yes	3 (6.25%)	1 (4%)	
No	45 (93.75%)	24 (96%)	

In group 1, the mean hospital stay was 11.5 days and the 30-day mortality rate was 6.25%. As the most feared complication after colorectal surgery, it was observed 12.5% of anastomotic fistulas and 12.5% of reoperations in this retrospective series.

In group 2, there was a three-day reduction in the length of hospital stay, a statistically significant result (eight days, p=0.002). There was also a decrease in the mortality rate (4%, p=0.56), anastomosis fistula (4%, p=0.23), and number of reoperations (8%, p=0.43), although without statistical significance. It is worth considering that these better outcomes occurred together with a significant reduction in the use of prophylactic abdominal drains (from 68.75% in group 1 to 16% in group 2, p<0.001) and in the rate of mechanical bowel preparation (87.5% to 16%, p<0.001). In this group, only one patient developed colonic fistula; he was doing good on the 3^rd^ postoperative day, was discharged from hospital, but was readmitted on the 6^th^ day with abdominal sepsis. He was reoperated and a dehiscence of the anastomosis in the left colon was observed. Peritoneal cleaning, resection of the affected area and completion of a Hartmann colostomy were performed, but the patient died, being the responsible for the mortality in this series.

It should be remembered that 92% of patients in the ERAS group were operated on for malignant colon and rectal neoplasms. The R0 resection rate was 100%, with the mean number of resected lymph nodes being 22 (15-42).

## DISCUSSION

In recent years, several centers of excellence have shown the feasibility of implementing the ERAS protocols, reporting improved results. In general, there is an earlier functional recovery of the patients, leading to a decrease in the length of hospital stay and generating cost savings.

Ota et al.^18^, in a multicenter study, evaluated the results obtained with the ERAS protocol in Japan. They observed that the postoperative oral diet was started earlier in the ERAS group, in addition to verifying a faster return of gastrointestinal function and reaching hospital discharge criteria much earlier than in the control group, all in a statistically significant way. It should be remembered that more than 90% of the operations were laparoscopic, and total compliance rate with the protocol was 84.7%. In our series, similar results were achieved even with a compliance rate of 68% and with 20% minimally invasive operations, which shows that improvements can still be implemented in order to achieve even better outcomes.

Laparoscopic colorectal surgery has been shown to be safe, with benefits related to the method (less postoperative pain, early mobilization, lower incidence of infectious and abdominal wall complications, among others) and with similar oncologic results^1^ ^,^ ^22^. However, it requires specific material for its adequate fulfillment, which is not available in the Brazilian public health system. Thus, in our series, the patients submitted to the minimally invasive operations were operated on using permanent material, and the anastomosis was made outside the peritoneal cavity through a small incision in which the surgical specimen was removed. This point was the main responsible for the low intraoperative compliance rate to the protocol, and requires funding from the Unified Health System (SUS) to correct this deficiency.

A Canadian study published in 2016 showed that, with a 60% compliance rate to the ERAS protocol, it was possible to reduce hospitalization time by 1.5 days (p<0.0001), in addition to reducing the overall rate of complications, especially pulmonary ones, by 11.9%. Besides that, there was a large cost saving per patient, ranging from $ 2,668 to $ 5,643^17^. In our series, the main infectious complications were reduced (anastomosis fistulas and abdominal abscesses), in addition to a three-day reduction in the length of hospital stay.

Similar results were verified in a US study conducted by Thiele^21^ in 2015, which evaluated 109 consecutive patients managed through the ERAS protocol. The authors achieved a significant reduction in length of hospital stay, overall complication rate, and costs. It is concluded, therefore, that the protocol is reproducible in different scenarios, allowing equally satisfactory outcomes.

Analyzing the death observed in our series, there are some possibilities for its occurrence. Perhaps the main reason was that there should be follow-up and/or daily telephone contact with patients who are discharged very early, in order to identify and promptly allow the treatment of those who develop infectious complications. We still deal with patients with low intellectual level in Brazilian SUS, which implies an active search by the care team in an attempt to detect postoperative complications. The other option would be to select patients who could be followed on an outpatient basis, and those who should remain for a longer period of observation inside the hospital.

Three important points deserve a brief comment, since they refer to true dogmas in colorectal surgery.

The first one is early oral feeding in the postoperative period. The literature is unanimous in demonstrating that this practice does not induce fistulas’ formation nor dehiscences, besides bringing comfort to patients, restoring the physiology of the gastrointestinal tract and even contributing to the sooner return of its functioning^8^ ^,^ ^12^.

The second is the use of prophylactic drains in the abdominal cavity after colorectal surgery. The fear of the occurrence of fistulas or collections in the postoperative period of these patients diffused, among surgeons, the practice of prophylactic peritoneal drainage. However, several studies in recent years have found that drains do not reduce the occurrence of fistulas, nor the incidence of abdominal collections or reoperations^9^ ^,^ ^19^. In this series, omitting the use of drains did not increase the incidence of these complications; on the contrary, mortality rate and the occurrence of fistulas were reduced in the ERAS group. It should be recalled that of the six patients reoperated in group 1, five (83.3%) had abdominal drains. In addition, of the three that evolved to death in this same group, two (66.6%) had prophylactic drains.

The third topic, still controversial, concerns preoperative bowel preparation. When used alone, several studies have shown that the mechanical preparation with laxatives, in order to reduce the amount of residues, showed no benefit in reducing infectious complications and mortality, compared to patients in whom this preparation was not performed^6^ ^,^ ^15^. This result was observed in this study. However, some recent papers have brought to the foreground the association of colonic mechanical preparation with enteral antibiotics aiming to reduce colonic bacterial flora. This association, in these studies, has been shown to decrease the occurrence of surgical site infections and abdominal collections due to anastomoses fistulas^3^ ^,^ ^11^. The recommendation of the ERAS Society (conducted prior to publication of these results) is opposed to the mechanical preparation, but does not address its association with antibiotics. It is possible that this topic will be reviewed in future protocol updates.

This paper has some limitations. First, the use of retrospective data for group 1 assessment limits the quality of these results. In this group of 48 patients, the operations were performed by two different teams, which denote a global result for the institution, but interfere with their comparison with group 2, in which only a specialized team was responsible for the operations. In addition, there are still a small number of cases, despite being fairly controlled and prospective. In this way, it is necessary to wait for the outcomes in a more representative population, so that the real size of the changes can be verified.

## CONCLUSION

The implementation of the ERAS protocol is feasible and beneficial for health institutions and patients, bringing on advances in care. Looking for a better compliance to the recommendations proposed by these guidelines should be the way to improve outcomes. Paradigm shifts for better results.
